# Giant enhancement of nonreciprocity in gyrotropic heterostructures

**DOI:** 10.1038/s41598-023-48503-9

**Published:** 2023-12-11

**Authors:** Ioannis Katsantonis, Anna C. Tasolamprou, Thomas Koschny, Eleftherios N. Economou, Maria Kafesaki, Constantinos Valagiannopoulos

**Affiliations:** 1 Institute of Electronic Structure and Laser, Foundation for Research and Technology Hellas, 70013 Heraklion, Greece; 2 Department of Material Science and Technology, University of Crete, 70013 Heraklion, Greece; 3 Department of Physics, National and Kapodistrian University of Athens, 15772 Athens, Greece; 4 Ames National Laboratory, Ames, IA 50011 USA; 5 Department of Physics and Astronomy, Iowa State University, Ames, IA 50011 USA; 6 Department of Physics, University of Crete, 70013 Heraklion, Greece; 7 School of Electrical and Computer Engineering, National Technical University of Athens, 15772 Athens, Greece

**Keywords:** Engineering, Physics, Sensors and biosensors, Materials science, Metamaterials

## Abstract

Nonreciprocity is a highly desirable feature in photonic media since it allows for control over the traveling electromagnetic waves, in a way that goes far beyond ordinary filtering. One of the most conventional ways to achieve nonreciprocity is via employing gyrotropic materials; however, their time-reversal-symmetry-breaking effects are very weak and, hence, large, bulky setups combined with very strong magnetic biases are required for technologically useful devices. In this work, artificial heterostructures are introduced to enhance the effective nonreciprocal behavior by reducing the contribution of the diagonal susceptibilities in the collective response; in this way, the off-diagonal ones, that are responsible for nonreciprocity, seem bigger. In particular, alternating gyrotropic and metallic or plasmonic films make an epsilon-near-zero (ENZ) effective-medium by averaging the diagonal permittivities of opposite sign, representing the consecutive layers. The homogenization process leaves unaltered the nonzero off-diagonal permittivities of the original gyrotropic substance, which become dominant and ignite strong nonreciprocal response. Realistic material examples that could be implemented experimentally in the mid-infrared spectrum are provided while the robustness of the enhanced nonreciprocity in the presence of actual media losses is discussed and bandwidth limitations due to the unavoidable frequency dispersion are elaborated. The proposed concept can be extensively utilized in designing optical devices that serve a wide range of applications from signal isolation and wave circulation to unidirectional propagation and asymmetric power amplification.

## Introduction

Electromagnetic nonreciprocity is an acclaimed feature manifested in systems where the response depends on the direction of the electromagnetic waves flow. In such configurations, the position of a source and a receiver are not interchangeable in the sense that the fields created by a source at the receiver are different from them developed when the source is placed at the position of the receiver and vice versa^1–3^. Nonreciprocity facilitates unidirectional propagation rendering the respective components crucial for the efficient operation in the majority of electromagnetic setups calling for complete control of signals flow; as a result, the related research attracts growing attention^4^. Relevant devices involve, among others, source protectors from unwanted reflections^5,6^, microwave circulators and isolators^7–10^, leaky-wave and phased-array antennas^11,12^, optical isolators^13,14^, photonic diodes^15^, and one-way flat lenses^16^.

Magneto-optical (MO) materials, like ferrites that exhibit anisotropic electromagnetic properties controlled by a magnetic force, are usually required to induce nonreciprocity. Applying externally a static magnetic field, breaks the time-reversal symmetry and leads to the desired unidirectional propagation of waves^17–21^. However, the magneto-optical effect in commonly used materials is usually weak and, thus, achieving considerable nonreciprocity for practical implementations calls for a bulky MO structure and high magnetic biases. Therefore, there is a significant effort towards either enhancing the nonreciprocal response into such a material or resorting in magnetic-free alternatives. Recently, the latter approach has employed nonlinear systems^22–26^, active configurations with Parity-Time symmetry^27–30^, circuits with spatio-temporal modulation^15,31–37^, magneto-optical configurations with hyperbolic metamaterials^38–41^ or Weyl semimetals^42^.

To enhance and manipulate the spontaneous magneto-optical response in materials and further boost the natural nonreciprocity, a variety of complex structures have been proposed. Gyrotropic photonic crystals, i.e. periodic structures with controllable bandgaps, have been early shown to sustain nonreciprocal, unidirectional waves paving the way to the development of the topological photonics realm^43–46^. In particular, metamaterials consisting of coated nanorods have been shown to support nonreciprocal light transmission^47^, ring resonator integrating ferrites can exhibit enhanced optical isolation^48^ while nanodisk arrays may host amplified MO effect^49^. On the other hand, simpler structures aiming to manipulate the range of the operation and boost the nonreciprocity can involve planar dielectric and chiral/magneto-optical multilayer systems^21,50^.

Inspired by these developments, this work examines a scheme for extensively enhancing the weak response of natural magneto-optical materials. In particular, we use gyrotropic multilayers which are characterized by poor nonreciprocity as indicated by the small magnitude of the off-diagonal elements of their permittivity tensor. However, if we incorporate them together with a negative-epsilon background host to form an average effective medium, the diagonal elements of the effective permittivity tensor of the latter can be designed to become close to zero while maintaining the magnitude of the off-diagonal elements; thereby, the gyrotropic response of the equivalent structure is expected to get hugely amplified. Our analytical calculations verify the proposed concept and show that these gyrotropic/plasmonic multilayers can exhibit giant nonreciprocity compared to the respective bulk gyrotropic structures of the same size. Importantly, the field distribution across the layers changes substantially depending on from which side we excite the structure. Effective medium approximation and numerical simulations validate our analytical results. We also observe this substantial enhancement even in the presence of losses and when using actual media with realistic dispersion in the mid-infrared spectrum. The herein demonstrated unidirectional propagation of circularly polarized waves leading to almost perfect optical isolation, is a prerequisite for multiple photonic operations concerning spin-polarized optical signal processing.

## Theory background

### Gyrotropic media

One of the first recorded manifestations of nonreciprocity was the Faraday rotation of linearly polarized light through a rod of glass under an external magnetic field; indeed, the rotation angle is reversed once the direction of incident wave or that of the magnetic bias, gets flipped. As a result, the electrons into the material are not only affected by the alternating electric field application but also participate in an effective circular motion and, subsequently, acquire different microscopic polarizabilities from those obtained in the absence of static magnetic bias. Such a property of a homogeneous gyrotropic material is characterized by the effective relative permittivity tensor:

1 $$\begin{aligned}{}[\varepsilon ]= \left[ \begin{array}{ccc} \varepsilon _t &{} -i \varepsilon _g &{} 0 \\ i\varepsilon _g &{} \varepsilon _t &{} 0 \\ 0 &{} 0 &{} 1 \end{array}\right] , \end{aligned}$$

expressed in a Cartesian coordinate system (*x*, *y*, *z*), for an external magnetic field along *z* direction. Such a material is called gyrotropic or magneto-optical (MO) and can be modeled by a diagonal permittivity $\varepsilon _t$, indicating the ordinary dielectric function, accompanied by two opposite off-diagonal permittivities $(\pm i \varepsilon _g)$ characterizing the nonreciprocal response of the medium. In the lossless scenario, we have purely real (diagonal) or purely imaginary (off-diagonal) values for the permittivity elements, namely, $\varepsilon _t,\varepsilon _g\in \mathbb {R}$.

In an unbounded gyrotropic medium, a circularly polarized (CP) plane electromagnetic wave traveling in $+z$ direction propagates in the same manner as in an isotropic medium with equivalent permittivity $\varepsilon _{\pm }=\varepsilon _{t} \pm \varepsilon _{g}$, and its electric field can be written as: ${\textbf {E}}_{\pm }=C_{\pm }(\hat{{\textbf {x}}} \pm i\hat{{\textbf {y}}})e^{i k^{\pm } z}$, where $k^{\pm }=k_0 \sqrt{\varepsilon _{t} \pm \varepsilon _{g}}$ are the respective wavevectors, $k_0=\omega /c=2\pi /\lambda$ is the free-space wavenumber, $\omega$ the angular frequency of operation, $\lambda$ and *c* the wavelength and the speed of light into vacuum. We suppressed the explicit factor $e^{-i\omega t}$ for the harmonic time dependence throughout the paper. The corresponding magnetic fields can be determined by ${\textbf {H}}_{\pm }= \mp \frac{i}{Z^{\pm }} {\textbf {E}}_{\pm }$, where $Z^{\pm }=\eta _0/\sqrt{\varepsilon _{t} \pm \varepsilon _{g}}$ is the wave impedance for right-handed circularly polarized (RCP, denoted by +) and left-handed circularly polarized (LCP, denoted by -) waves and $\eta _0$ is the wave impedance of free space. In other words, circularly polarized waves with opposite helicities experience different refractive indexes and different wave impedances; therefore, if one considers a single slab of gyrotropic material with finite thickness, the transmission coefficients of the field, $T_{\pm }$, are different for RCP and LCP waves^50^.

In gyrotropic media^3,50^, the transmission coefficient for RCP (LCP) waves propagating from the left to the right is identical to the transmission coefficient for LCP (RCP) waves propagating from the right to the left. Hence, the nonreciprocal effect can be quantified by $\Delta \tau =\tau _{+}-\tau _{-}$ where $\tau _{+}=|T_{+}|^2$ is the RCP transmitted power for RCP and $\tau _{-}=|T_{-}|^2$ is the LCP transmitted power for LCP waves incident from the same side. In the extreme scenario of a perfect optical isolator, one of the two transmissivities will vanish and, simultaneously, the other will be equal to unity (maximal nonreciprocity, $\Delta \tau =\pm 1$).

### Multilayered gyrotropic heterostructures

The bottleneck in emulating large nonreciprocity with MO structures lies in the small magnitude of the off-diagonal elements of permittivity tensor (1), namely, the fact that usually $|\varepsilon _{g}|\ll |\varepsilon _{t}|$. We advocate that a way to overcome this issue is employing homogenizable multilayers that incorporate MO materials accompanied by plasmonic media that exhibit a negative permittivity with magnitude as close as possible to $\varepsilon _t$ and effectively shrink the diagonal permittivities of the new structure.

**Figure 1:**
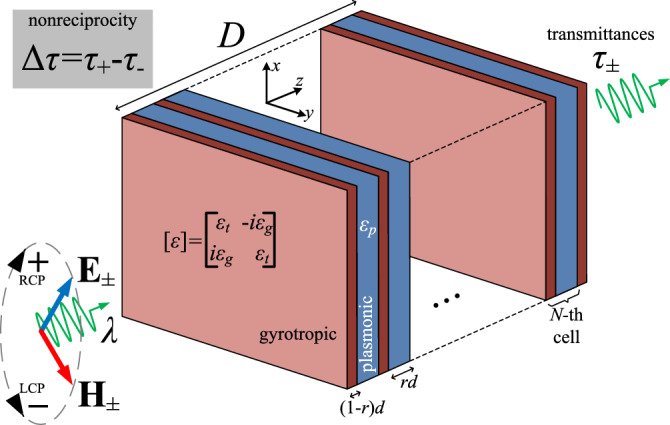
A multilayered structure of thickness *D* and *N* cells, each of which comprises a gyrotropic slab with relative permittivity tensor $[\varepsilon ]$ and a plasmonic slab with relative permittivity $\varepsilon _{p}$. The heterostructure is excited by a normally incident circularly polarized wave. Each cell has thickness *d* and the plasmonic layers have a filling factor $0<r<1$. For each of the directions of rotation of the incoming electromagnetic field $\{{\textbf {E}}_{\pm },{\textbf {H}}_{\pm }\}$ characterized either as RCP, (right-handed circular polarization, clockwise, subscript $+$) or LCP, (left-handed circular polarization, counter-clockwise, subscript −), the device produces transmissivities $\tau _{\pm }$. The difference $\Delta \tau \equiv \tau _{+}-\tau _{-}$ is a metric of how nonreciprocal is the structure.

To test and demonstrate the potential of this idea we consider the setup of Fig. 1. This heterostructure has an overall thickness *D* and comprises *N* cells. Each cell is a bilayer of size *d* containing a plasmonic film of filling factor $0<r<1$ and relative permittivity $\varepsilon _p$ and a gyrotropic film of thickness $(1-r)d$, characterized by the relative permittivity tensor (1). Note, though, that $D=(N+1-r)d$, since we have deliberately added a gyrotropic layer at the rear boundary of the structure to render it symmetric ($(1-r)$ is the MO material filing ratio within the unit cell); in this way, the reflection from both sides is of equal strength ($|R_{+}|^2=|R_{-}|^2$) and the only difference in the response $\Delta \tau$ concerns the related nonreciprocity.

The setup is investigated under normal illumination of circularly polarized electromagnetic waves $\{{\textbf {E}}_{\pm },{\textbf {H}}_{\pm }\}$ either right-handed (RCP, subscript $+$) or left-handed (LCP, subscript −), with operational wavelength $\lambda$. In particular, the electric field components of incoming waves are of unitary amplitude and take the form ${\textbf {E}}_{\pm }^\text{inc}(z)= (\hat{{\textbf {x}}} \pm i \hat{{\textbf {y}}})e^{i k_0 z}$. In this case, the transmitted ($z>D$) and reflected ($z<0$) electric fields are given by:

2 $$\begin{aligned} {\textbf {E}}_{\pm }^\text{tran}(z) = T_{\pm }(\hat{{\textbf {x}}} \pm i \hat{{\textbf {y}}})e^{i k_0 z}~~~~~~~,~~~~~~~ {\textbf {E}}_{\pm }^\text{ref}(z) = R_{\pm }(-\hat{{\textbf {x}}} \pm i \hat{{\textbf {y}}})e^{-i k_0 z}, \end{aligned}$$

where $T_{\pm }$ , $R_{\pm } \in \mathbb {C}$ are the complex transmission and reflection coefficients, respectively. These quantities are rigorously determined by implementing the transfer-matrix approach and imposing the necessary boundary conditions in each structure, as described in the "Methods" section.

If one puts slices of the gyrotropic material of (1) with $\text{Re}(\varepsilon _{t})>0$ into a “sea” with isotropic plasmonic medium having $\text{Re}(\varepsilon _{p})<0$, the diagonal elements of the overall effective permittivity matrix will be suppressed and, thus, the nonreciprocity expressed via $\varepsilon _{g}$ will get artificially boosted in the multilayered setup. In other words, we propose a new material, comprising layers of a gyrotropic substance, which is expected to be much more nonreciprocal compared to its basic ingredient, just by filling the gaps with an ordinary reciprocal metal, given the fact that $\text{Re}(\varepsilon _{t})\text{Re}(\varepsilon _{p})<0$.

In most of the examined cases, we consider lossless media $(\text{Im}(\varepsilon _{t})=\text{Im}(\varepsilon _{p})=\text{Im}(\varepsilon _{g})=0)$ and equal volume filling factors for both media $(r=1/2)$, in an attempt to limit the dimensions of parametric space by ignoring non-critical quantities. Importantly, we will work around the frequency that $\text{Re}(\varepsilon _{t})\cong -\text{Re}(\varepsilon _{p})>0$, where maximal nonreciprocity enhancement is expected; on the other hand, realistic (and, therefore, small) values for the nonreciprocity for MO materials will be used, such as $0<\text{Re}(\varepsilon _{g})/\text{Re}(\varepsilon _{t})<0.01$. The transmissivities $\tau _{\pm }$ and the nonreciprocity metric $(\Delta \tau )$ will be mainly represented with respect to the thickness of the structure, *D*, normalized by the operational wavelength $\lambda$. In this way, one may record how long the layered device should be to achieve high scores (close to unity) for the nonreciprocity indicator $\Delta \tau$. Moreover, by inspection of the graphs $\tau _{\pm }=\tau _{\pm }(D/\lambda )$ or $\Delta \tau =\Delta \tau (D/\lambda )$, the frequency response of a device with fixed *D* can be understood.

## Results

### Nonreciprocity at a single gyrotropic layer

First of all, we will discuss under which conditions a single homogeneous gyrotropic layer may have a strongly nonreciprocal response under illumination of circularly polarized waves with different helicities. As mentioned above, these eigenwaves “feel” the background medium differently since the permittivity $\varepsilon _{\pm }=\varepsilon _{t} \pm \varepsilon _{g}$ is dependent on the type of circular polarization. Naturally, the maximum spread between the two responses is achieved when these two complex quantities are as different as possible, namely, when $\varepsilon _{t}$ approaches zero (epsilon-near-zero medium, ENZ^51^). In particular, when $\varepsilon _{t}=0$, one obtains $\varepsilon _{-}=-\varepsilon _{g}$ so that the gyrolayer becomes opaque to LCP waves and transparent to RCP waves since $\varepsilon _{+}=\varepsilon _{g}>0$. Therefore, in the lossless case, it is expected that RCP waves are tunneled through the gyrolayer with unitary transmission by tuning the thickness of the slab *D* so that $k_{+}=l\pi /D$, $l\in \mathbb {N}$ (Fabry-Perot resonances) at the desired frequency range.

**Figure 2:**
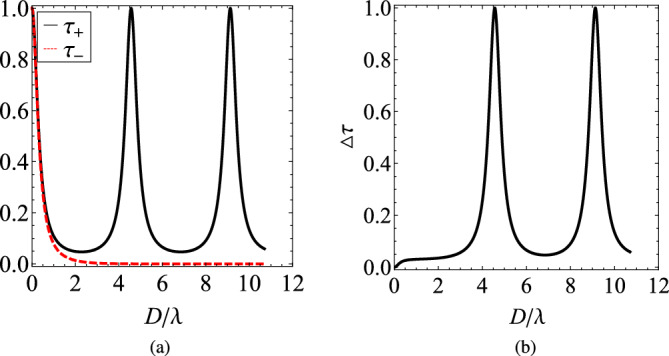
(**a**) The transmissivities $\tau _{\pm }$ as functions of the thickness of the gyrolayer *D* normalized by the operational wavelength $\lambda$. (**b**) The difference in the transmissivities $\Delta \tau$ as function of $D/\lambda$. Plot parameters: $\varepsilon _{t}=0$, $\varepsilon _{g}=0.012$.

To demonstrate this idea, we represent in Fig. 2a the transmissivities $\tau _{+}$ and $\tau _{-}$ as functions of $D/\lambda$ in an ENZ gyrolayer with $\varepsilon _{t}=0$ and $\varepsilon _{g}=0.012$, which is a realistic value for common MO materials^52^. Apparently, the response is oscillating unboundedly when the wave sees the material as lossless dielectric (RCP) and decays with the cavity size when the medium behaves as lossless plasmonic (LCP). In Fig. 2b, where the difference $\Delta \tau$ between the two transmissivities is shown, we observe a perfect discrimination ($\Delta \tau =1$) between the two polarization states around $D/\lambda \cong 4.55$ (first Fabry-Perot resonance) and $D/\lambda \cong 9.10$ (second Fabry-Perot resonance). However, naturally homogeneous ENZ media ($\varepsilon _t=0$) are particularly challenging to be attained, especially if low (or zero, as in the elaborated example) losses are required; that is why, in the following, we propose a different route to obtain qualitatively similar results from a structured material.

### Giant enhancement of nonreciprocity

**Figure 3:**
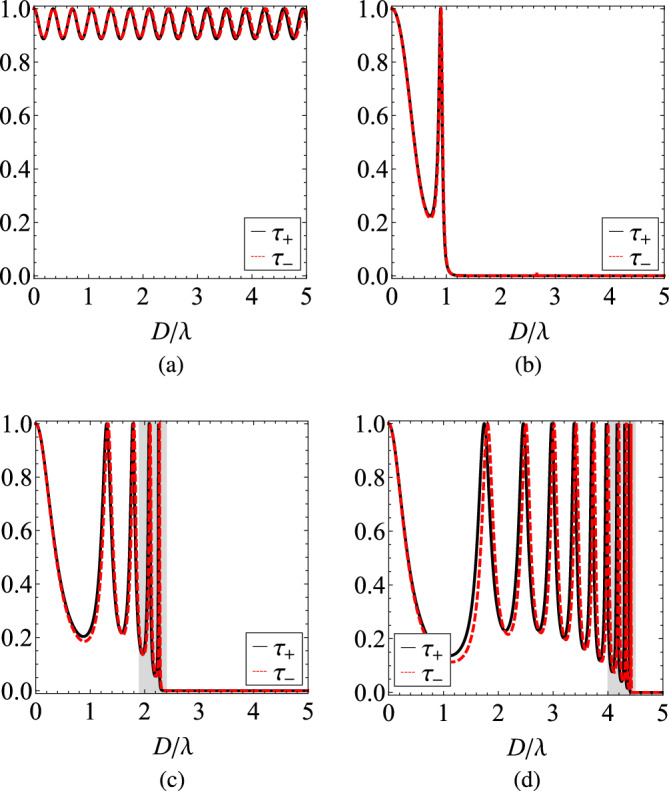
The transmissivities $\tau _{\pm }$ as functions of the overall thickness of the setup *D* normalized by the operational wavelength $\lambda$ for various numbers of cells *N*: (**a**) a single gyrotropic slab, (**b**) $N=2$, (**c**) $N=5$, (**d**) $N=10$. In the last two Figures, the shaded regions remark the ranges of $D/\lambda$, at which the transmissivities exhibit large variability. Plot parameters: $r=1/2$, $\varepsilon _{p}=-2$, $\varepsilon _{t}=2$, $\varepsilon _{g}=0.012$.

In order to emulate an effective zero index material, we consider and analyze the nonreciprocal response of a multilayered heterostructure consisting of gyrotropic/plasmonic bilayers, as the one depicted in Fig. 1. In Fig. 3, we show the transmissivities $\tau _{\pm }$ as functions of the optical thickness $D/\lambda$ of the structure for various number *N* of cells with size ($d=D/(N+1-r)\cong D/N$). Obviously, $\tau _{+}=\tau _{-}=1$ for $D/\lambda \rightarrow 0$, regardless of the number of layers *N* since the whole setup is infinitesimally thin and, thus, fully transparent. It is also clear that in the absence of plasmonic layers (Fig. 3a), a single gyrotropic slab treats the two polarizations in a similar way and transmits almost $100\%$ of the incoming power, with small oscillations for increasing $D/\lambda$, due to Fabry-Perot interference. Note that this is not the case in Fig. 2, where an unrealistic $\varepsilon _t=0$ is assumed. In addition, the phase difference between the two curves $\tau _{\pm }$, owing to different propagating modes $k_{\pm }$, becomes greater for larger $D/\lambda$. However, in order for the metric $|\Delta \tau |$ to take values comparable to unity, a huge thickness $D/\lambda$ is necessary.

The situation changes dramatically in Fig. 3b when just two plasmonic layers ($N=2$) are inserted; in particular, the transmitted power exhibits a sharp maximum close to $D\cong 0.9\lambda$ and, beyond that point, a bandgap appears dictating a $100\%$ reflection. Once the number of cells *N* increases (Fig. 3c and d), more maxima in the transmissivities $\tau _{\pm }$ emerge, which get more abrupt for thicker designs. These highly selective responses provide a fertile ground for strong nonreciprocity since the slightest shift may lead to substantial differences $\Delta \tau$ across narrow wavelength bands.

**Figure 4:**
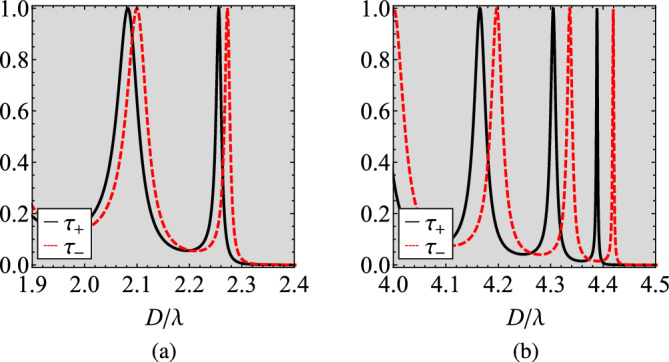
Detail of two last Figures of Fig. 2, across the shaded regions of large variability. (**a**) $N=5$, (**b**) $N=10$. Same plot parameters as in Fig. 3.

In Fig. 4, we show certain parts in detail from Fig. 3c and d where $N=5$ and $N=10$ cells are assumed respectively. More specifically, we regard the shaded bands just below the bandgap, where the highest density of states appears and significant response variability is recorded. In Fig. 4a, we clearly note that the spread between the two transmissivities $\tau _{\pm }$ opens in the vicinity of the thicknesses *D* that they are both maximized; indeed, a tiny dissimilarity between the curves leads to giant difference close to their sharp resonances. The same conclusions hold for Fig. 4b: the number of emerged peaks increase with the number of layers while their selectivity gets boosted with the thickness *D* of the structure. Especially for $D\cong 4.4\lambda$, $\tau _{-}$ is minimized and simultaneously $\tau _{+}$ gets maximized, giving a $|\Delta \tau |$ close to unity.

**Figure 5:**
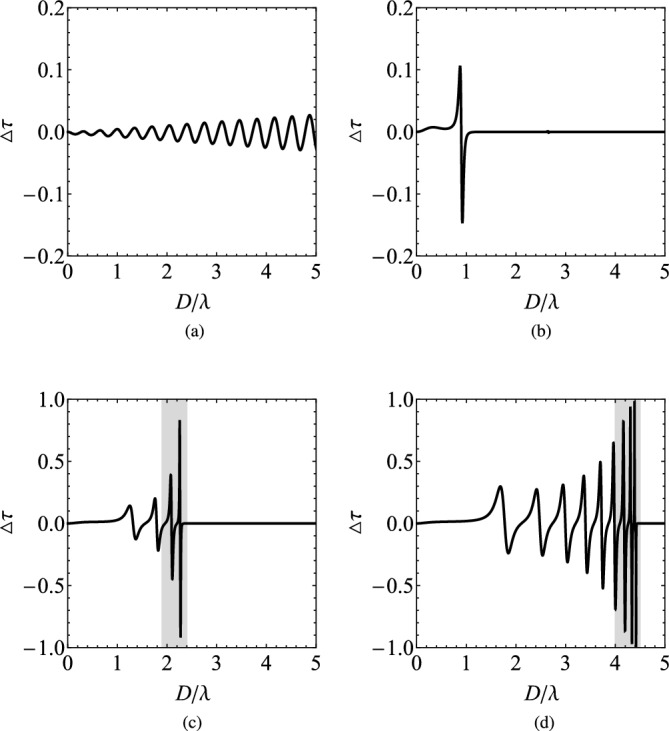
The difference in the transmissivities $(\Delta \tau )$, indicating the nonreciprocity of the device, as function of $D/\lambda$ for exactly the same cases of Fig. 3: (**a**) a single gyrotropic slab, (**b**) $N=2$, (**c**) $N=5$, (**d**) $N=10$. Again, in the last two Figures, shaded regions indicate bands of $D/\lambda$, at which the represented quantity exhibits large variability. Same plot parameters as in Fig. 3.

In order to characterize the nonreciprocal response of our system and, through it, its potential utility as optical isolator, we further analyze the scattering properties, calculating the difference in transmission $\Delta \tau =\tau _{+}-\tau _{-}$. The setups investigated in Fig. 3 are again examined and we notice that, for a single MO slab of thickness *D* (Fig. 5a), the metric $\Delta \tau$ fluctuates around zero level with a negligible amplitude that increases reluctantly with the size of the gyrolayer. In Fig. 5b, where we assume $N=2$, we notice a significant change in $\Delta \tau$ from high positive to even larger negative values at $D\cong 0.9\lambda$, as expected from Fig. 2b. It is remarkable that, compared to the nonreciprocal performance of a gyrotropic layer, there is an enhancement in $\Delta \tau$ by more than ten times, indicating a substantial improvement in nonreciprocity simply by using a couple ($N=2$) of cells.

In Fig. 5c, we increase the number of layers ($N=5$) and, as indicated by Figs. 3c, 4a, multiple Fabry-Perot interferences happen and several double resonances for $\Delta \tau$ occur, corresponding to abrupt maximization in $\tau _{\pm }$ at very similar thicknesses *D*. As mentioned above, not only the height but also the sharpness of peaks increases with *D* and a giant amplification of nonreciprocity (compared to the plasmonic-free scenario) is recorded for $D\cong 2.2\lambda$. The enhancement in $\Delta \tau$ is even more remarkable in Fig. 5d at which $N=10$ (as in Figs. 3d, 4b); indeed, a combination of multiple MO and metallic layers can amplify $\Delta \tau$ by at least two orders of magnitude in comparison with a single gyrotropic piece of identical size *D*.

Another point to be taken into account is that in the stacked heterostructure, the overall thickness of MO material is not *D* but less, namely, close to $(1-r)D$ where *r* is the filling factor of the metal. Therefore, if the same volume of MO substance in the single gyrotropic slab is incorporated in the layered system of Fig. 1, it will increase its overall thickness *D*. In other words, the enhancement in $\Delta \tau$ is more pronounced when the same amount from the gyrotropic medium is utilized.

**Figure 6:**
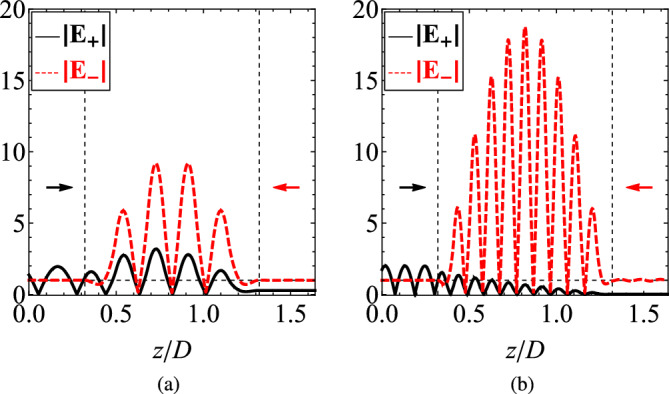
The magnitudes of the electric field $|{\textbf {E}}_{\pm }|$ represented across the longitudinal direction *z*/*D* when the structure is illuminated from different sides: $|{\textbf {E}}_{+}|$ is the signal when the left port is excited while $|{\textbf {E}}_{-}|$ expresses the response when the right port is excited (arrows indicate the side of illumination). The thickness of the setup is selected so that $|\Delta \tau |$ is maximized: (**a**) $N=5$, $D = 2.10\lambda$, (**b**) $N=10$, $D = 4.42\lambda$. Rest of plot parameters same as in Fig. 3.

As remarked above, the transmissivity of the device under LCP excitation from the left side (fields ${\textbf {E}}_{-}$) equals to its transmissivity under RCP excitation from the right side; therefore, the responses are just computed by illuminating the structure with RCP waves from its two opposite sides. In Fig. 6, we represent the magnitude of the electric fields $|{\textbf {E}}_{\pm }|$ across the normal-to-the-interfaces *z* axis of the regarded setup when being fed at the corresponding port. In Fig. 6a, we choose an optimal (giving maximum $| \Delta \tau |$, $D\cong 2.10\lambda$) design comprising $N=5$ cells. Once it gets illuminated from the leftmost side, strong reflections are recorded, a standing-wave pattern is formed into the multilayered layout and the transmission is suppressed. One can recognize the borders of the gyrolayers by identifying the peaks of the waveforms since, into lossless plasmonic media, only evanescent waves are developed. When the other (rightmost) port is on, the transmission is almost total which, inevitably, leads to a matching regime (zero reflection), due to the passivity of the device and the imposed conservation of energy. In this way, not only a completely different response $\tau _{\pm }$ is recorded but also the power into the layers changes dramatically even though the created standing waves possess the same antinodes.

In Fig. 6b, we regard a system with more cells ($N=10$, $D\cong 4.42\lambda$) that is also characterized by a large $|\Delta \tau |$. When the front boundary is illuminated (${\textbf {E}}_{+}$), the transmission is nullified, while in the case of rear-boundary excitation (${\textbf {E}}_{-}$), the reflection is almost totally suppressed. It becomes, therefore, apparent that our device is suitable for unidirectional propagation. The reported setups can be also used for sensing since the signal across the layers becomes substantially different when one changes the feeding side; in particular, they adopt direction-dependent properties that get stronger by increasing the number of layers. It should be also stressed that, internally to the device, the developed fields are more significant compared to the reflective/transmissive ones due to the larger (and different) local permittivities.

### Effective medium approximation

Our basic idea behind choosing the proposed heterostructure of Fig. 1 for increasing the nonreciprocity of MO layers has been based on a simple mixing rule (Effective Medium Approximation, EMA) of gyrotropic and plasmonic permittivities, based on Maxwell-Garnett effective medium description. It yields an effective permittivity tensor (for $r=1/2$):

3 $$\begin{aligned}{}[\varepsilon _\text{eff}] = \begin{bmatrix} \frac{\varepsilon _t+\varepsilon _p}{2} &{} -i\frac{\varepsilon _g}{2} &{} 0 \\ i\frac{\varepsilon _g}{2} &{} \frac{\varepsilon _t+\varepsilon _p}{2} &{} 0 \\ 0 &{} 0 &{} 1 \end{bmatrix}. \end{aligned}$$

**Figure 7:**
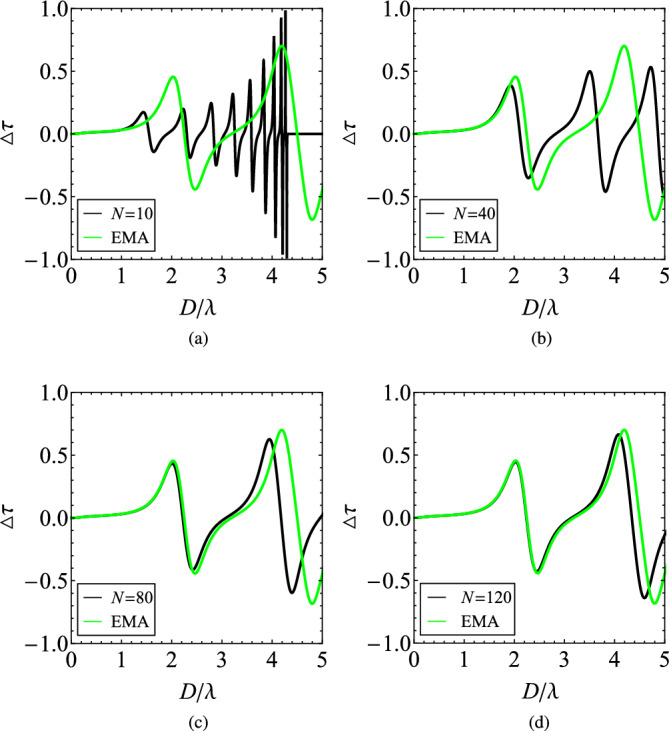
The difference in the transmissivities $\Delta \tau$, indicating the nonreciprocity of the device, as function of $D/\lambda$ for various numbers of cells *N* compared with the Effective Medium Approximation (EMA) given by (3). (**a**) $N=10$, (**b**) $N=40$, (**c**) $N=80$, (**d**) $N=120$. Plot parameters: $r=1/2$, $\varepsilon _p=-2$, $\varepsilon _{t}=2$, $\varepsilon _{g}=0.012$.

One may trivially solve the homogenized problem of a slab with thickness *D* filled with a medium characterized by (3) and represent the respective metric $\Delta \tau =\tau _{+}-\tau _{-}$ as a function of $D/\lambda$ in comparison with the one of the layered structure, as in Fig. 7. Similar results for the homogenized structure are depicted in Fig. 2. The permittivity $\varepsilon _{t}$ of the MO material is not taken exactly equal to ($-\varepsilon _{p}$) to avoid numerical issues involving the vanishing diagonal elements in (3). In Fig. 7a, we consider a MO/plasmonic layout with $N=10$ and realize that the two curves are totally different, especially when $D>\lambda$. On the contrary, in Fig. 7b, where $N=40$, the response of the structure coincides with the EMA curve for a more extensive interval of *D*; interestingly, the bandgap is pushed outside of the considered band of $D/\lambda$ due to the increased *N*. The deviation between the two sets of data (rigorous solution vs effective medium approximation) keeps closing when *N* gets larger and larger ($N=80$ in Fig. 7c and $N=120$ in Fig. 7d). The observed agreement between rigorous results and effective medium for substantial number of cells, in Fig. 7, justifies our choice of employing the considered configuration to amplify the effective nonreciprocity since the tensor (3) represents a nonreciprocal medium with strength proportional to $|\varepsilon _{g}|/|\varepsilon _{t}+\varepsilon _{p}|$. Not surprisingly, the effective medium approximation becomes a good description of the heterogeneous layered stack if the individual plasmonic layers become much smaller than the operational wavelength. Such a behavior is anticipated because the Maxwell-Garnett approach works well only in the quasistatic regime where the individual layer thicknesses are much smaller than the locally developed wavelengths. Note, additionally, that in the case of plasmonic layers, the effective medium description is applicable only if they are thinner than the metal skin depth so that the wave penetrates the interface. That is why we attain the nonreciprocity enhancement for an average ENZ effective medium with deeply subwavelength subdivisions (large *N*). It is stressed that more sophisticated formulas and more elaborate homogenization models are available to operate beyond the quasistatic limit^53,54^.

### Dissipation effect

**Figure 8:**
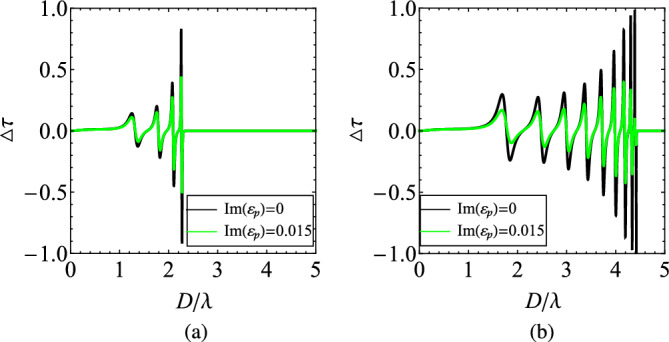
The difference in the transmissivities $\Delta \tau$, indicating the nonreciprocity of the device, as function of $D/\lambda$ in the presence of losses $\text{Im}(\varepsilon _p)\ne 0$: (**a**) $N=5$, (**b**) $N=10$. Same plot parameters as in Fig. 3.

In all the investigated scenarios so far, lossless designs have been employed; however, plasmonic media always host ohmic effects and thus is necessary to examine the dissipation influence on the effective nonreciprocity $\Delta \tau$ of the heterostructure. In Fig. 8, we represent the curves of $\Delta \tau =\Delta \tau (D/\lambda )$ with and without losses for two characteristic cases of Fig. 3. The shapes are similar but the peak-to-peak variation gets substantially shrunk since an amount of power is absorbed into the multiple metallic layers. That is why the appeared resonances become weaker in the presence of losses but their number and locations remain unaltered. Once again, we observe that the bandgap is only dependent on the number of layers and emerges at thicker designs *D* for increasing *N*. Finally, the sharper the maxima are, the more vulnerable to ohmic ($\text{Im}(\varepsilon _{p})\ne 0$) impacts get, due to their ultra-narrowband nature.

To mitigate the influence of losses on nonreciprocal response, one can apparently consider increasing the strength of the applied magnetic field; however, such an adjustment should take into account practical constraints and material limitations. Another possibility would be to increase the thickness of the MO material but this modification may render the layered system non-homogenizable and, thus, inapt to act as an effective medium. In addition, the utilization of metamaterials and photonic crystals can directly lead to the manipulation of the flow of light in ways that minimize losses and enhance nonreciprocal effects^55^. Lastly, incorporating gain media^56^ such as quantum wells, dye molecules or quantum dots, can always balance the aforementioned thermal dissipation.

### Materials dispersion

In all the previously examined cases, we have assumed that every layer is filled with materials behaving the same for all frequencies $\omega$. A more realistic version of our consideration may account for actual dispersive media like indium arsenide (InAs) which exhibits nonreciprocal response at THz regime in the presence of a magnetic bias. In particular, the relative permittivity tensor elements of doped InAs, according to (1), can be written as^57^:

4 $$\begin{aligned} \varepsilon _{t}(\omega )=\varepsilon _{\infty }+\frac{\omega _{p}^2 (\omega + i\Gamma )}{\omega [(\omega + i\Gamma )^2 -\omega _{c}^2]}~~~~~~~,~~~~~~~ \varepsilon _{g}(\omega )=\frac{\omega _{p}^2 \omega _{c}}{\omega [(\omega + i\Gamma )^2 -\omega _{c}^2]}, \end{aligned}$$

where $\omega _{p}^2=n e^2/(m \varepsilon _0)$ and $\omega _{c}=eB_0/m$ are the plasma and cyclotron frequencies, respectively. The notation $B_0$ is used for the static magnetic field while *e* is the electron charge. The effective mass of the particles into InAs equals to: $m=0.03 m_e$, where $m_e$ is the inertial electron mass. Note that the dielectric constant in the short wavelength limit is taken as $\varepsilon _{\infty }=12.3$, while *n* is the density of particles into the volume of the medium with a typical^58^ value of $n \cong 5.74\times 10^{18}~\mathrm{1/cm^3}$. In this way, the plasma and cyclotron frequencies are evaluated as: $\omega _{p}/(2\pi ) \cong 124.2~\text{THz}$ and $\omega _{c}/(2\pi ) \cong 1.4~\text{THz}$, for a feasible magnetic bias $B_0=1.5~\text{T}$. As far as the losses $\Gamma$ are concerned, they are taken equal to: $\Gamma /(2\pi )=0.3~\text{THz}$ based on experimental data for the scattering time of electrons^59^.

When it comes to the plasmonic media, ordinary metals are unsuitable since they become lossy at mid-infrared band and possess too high plasma frequencies, calling for unrealistically thin layers to achieve a vanishing effective average. Given the fact that InAs with a different plasma frequency $\omega '_{p}/(2\pi ) \cong 105.9~\text{THz}$ (corresponding to a lower charged carrier density $n'\cong 4.17\times 10^{18}~\mathrm{1/cm^3}$) acquires negative diagonal permittivity in the considered frequency regime, we decided to employ it as a plasmonic material too ($\text{Re}[\varepsilon '_t]=\text{Re}[\varepsilon _p]<0$). Indeed, plasma frequency is easily controlled via electron doping. It is stressed that the second MO medium is also regarded under magnetic bias $B_0$ since otherwise a potential fabrication of the setup will get very challenging requiring abrupt spatial variation for the applied static magnetic field. As a result, both layers of the unit cell get anisotropic, where the plasmonic one is characterized by diagonal $\varepsilon _p=\varepsilon _t'\ne \varepsilon _t$ and off-diagonal ($\varepsilon _g'\ne \varepsilon _g$) permittivities also defined in (4).

**Figure 9:**
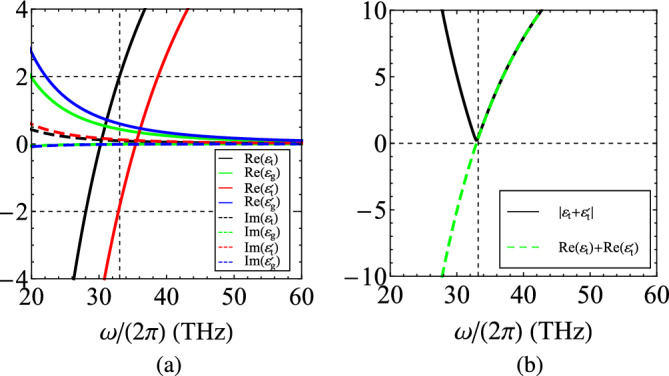
(**a**) The frequency variation of real and imaginary parts of the relative permittivities $(\varepsilon _t,\varepsilon _g)$ and $(\varepsilon _t'=\varepsilon _p,\varepsilon _g')$ characterizing the two InAs-based media (one of which plays the plasmonic role) incorporated at each unit cell, across the mid-infrared band. (**b**) The quantities $|\varepsilon _t+\varepsilon '_t|=|\varepsilon _t+\varepsilon _p|$ and $\text{Re}(\varepsilon _t)+\text{Re}(\varepsilon '_t)=\text{Re}(\varepsilon _t+\varepsilon _p)$ indicating what is the frequency around which the proposed concept can be best applied. Plot parameters: $\varepsilon _{\infty }=12.3$, $\omega _{p}/(2\pi ) \cong 124.2~\text{THz}$, $\omega _{c}/(2\pi ) \cong 1.4~\text{THz}$, $\omega '_{p}/(2\pi ) \cong 105.9~\text{THz}$, $\Gamma /(2\pi )= 0.3~\text{THz}$.

In Fig. 9a, we show the frequency dependence of the real and imaginary parts for the four permittivities $\left\{ \varepsilon _t,\varepsilon _g,\varepsilon _t',\varepsilon _g'\right\}$ across the investigated band. The real parts of the diagonal permittivities are increasing functions of frequency but cross the horizontal axis at different points. In particular, one directly observes that at $\omega /(2\pi )\cong 33.2~\text{THz}$, denoted by a vertical dashed line, the permittivity $\varepsilon _t$ of the dielectric gyrolayer has a real part with $\text{Re}(\varepsilon _t)=2$ while, for the plasmonic one, we receive $\text{Re}(\varepsilon _t')=-2$. In this way, the following results are referring locally (in the vicinity of frequency $\omega$) to the dispersive analog to the regime examined in most of the aforementioned examples. It is remarked that losses are small but non-negligible in both of the layers $\text{Im}(\varepsilon _t)\text{Im}(\varepsilon _t')\ne 0$. In Fig. 9b, we represent the quantity $|\varepsilon _{p}+\varepsilon _{t}|$, where $\varepsilon _p=\varepsilon '_t$ at mid-infrared frequencies; such a quantity indicates how suppressed the diagonal susceptibilities of (3) get and, accordingly, how big the (noreciprocal) off-diagonal elements look compared to them. It is clear that $|\varepsilon _{p}+\varepsilon _{t}|=|\varepsilon '_{t}+\varepsilon _{t}|$ gets minimized for $\omega /(2\pi )\cong 33.2~\text{THz}$. Similarly, the sum of the real parts $\text{Re}(\varepsilon _{p}+\varepsilon _{t})=\text{Re}(\varepsilon '_{t}+\varepsilon _{t})$ vanishes at the same frequency and, thus, the respective range offers the opportunity to test the introduced idea, in the presence of dispersion. Interestingly, the off-diagonal permittivities are of tiny magnitudes $|\varepsilon _g|,|\varepsilon _g'|<1$ for the major part of the regarded band; therefore, the proposed concept for nonreciprocity enhancement becomes, once more, well-justified.

**Figure 10:**
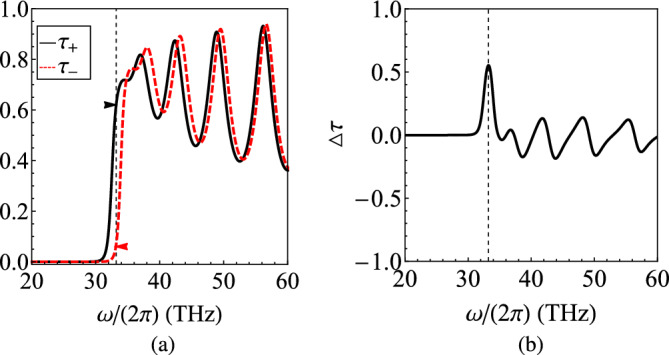
(**a**) The transmissivities $\tau _{\pm }$ as function of mid-infrared operational frequency $\omega /(2\pi )$ for InAs-based dispersive media. (**b**) The respective nonreciprocity indicator $\Delta \tau$. Plot parameters: $N=15$, $d=150~\text{nm}$, $r=1/2$. Materials same as those in Fig. 9.

In Fig. 10, we consider $N=15$ cells each of which has size of $d=150~\text{nm}$ and equal portions ($r=1/2$) from both media. In Fig. 10a, we notice that both transmissivities $\tau _{\pm }$ are vanishing for low frequencies since both materials are plasmonic and, thus, electromagnetically opaque. However, for $\omega /(2\pi )>30~\text{THz}$, they exhibit a similar oscillating behavior around substantial values. The small phase shift between the two curves creates a huge difference as indicated by the respective markers at $\omega /(2\pi )\cong 33.2~\text{THz}$. Such a feature is demonstrated in Fig. 10b where the frequency variation of $\Delta \tau$ is shown. Indeed, the difference in the response gets maximized at the aforementioned frequency and then fluctuates around zero point by taking moderate negative and positive values.

**Figure 11:**
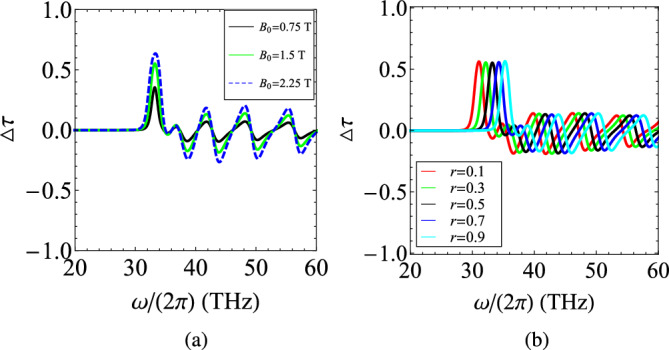
The metric $\Delta \tau \equiv \tau _{+}-\tau _{-}$ as function of mid-infrared oscillating frequency $\omega /(2\pi )$ for various: (**a**) magnetic biases $B_0$, (**b**) plasmonic filling factors *r*. Same parameters as in Fig. 9.

In Fig. 11a, we sketch the metric $\Delta \tau =\Delta \tau (\omega )$ for various magnetic biases $B_0$. It is clear that higher peaks appear and the nonreciprocal character of our heterostructure gets stronger for increasing $B_0$; that was anticipated since the cyclotron effect into both layers becomes amplified. It is additionally noteworthy that the peak in $\Delta \tau$ can be shifted across frequency axis once the filling factor *r* is being regulated as in Fig. 11b. This is also a natural outcome since the frequency at which the desired condition $\text{Re}(\varepsilon _t+\varepsilon _p)=0$ is satisfied, changes due to the asymmetry of the unit cell. That is another feature indicating that the elaborated idea for boosting the nonreciprocity is valid, regardless of the volume analogy between the two employed materials. Importantly, the controllability of the nonreciprocal resonances via parameters like $B_0$ and *r* demonstrates the potential of the effect to be tunable and reconfigurable in proportion to the application framework.

**Figure 12:**
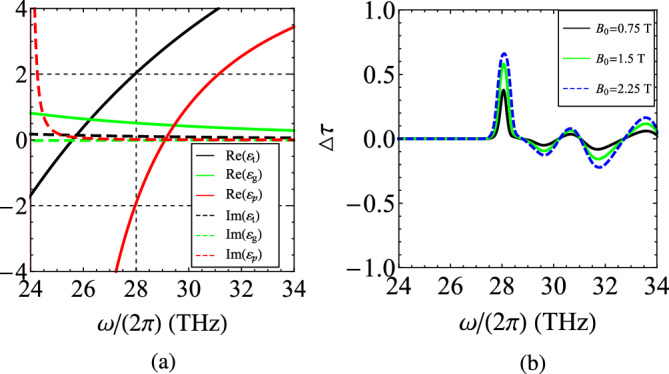
(**a**) The frequency variation of real and imaginary parts of the relative permittivities $(\varepsilon _t,\varepsilon _g)$ and $\varepsilon _p$ when the role of plasmonic material is played by SiC, across the mid-infrared band. (**b**) The nonreciprocity metric $\Delta \tau$ as function of operational frequency $\omega /(2\pi )$ for several magnetic biases $B_0$. Plot parameters: $N=20$, $d=200~\text{nm}$, $r=1/2$.

Alternatively, the role of the plasmonic substance can be played by materials like silicon carbide (SiC), just above the phonon-polariton resonance, which exhibit particularly low losses and, thus, are practically unaffected by the application of magnetic bias. The permittivity of SiC in this region is approximated by the formula^60^:

5 $$\begin{aligned} \varepsilon _p(\omega )=\varepsilon _{\infty }\left[ 1+\frac{\omega _{L}^2-\omega _{T}^2}{\omega _{T}^2-\omega ^2-i\omega \Gamma }\right] , \end{aligned}$$

where $$\varepsilon _{\infty }\cong 12.3$$, $$\omega _L/(2\pi )\cong 29.1~\text{THz}$$, $$\omega _T/(2\pi )\cong 23.9~\text{THz}$$ and $$\Gamma /(2\pi )\cong 0.014~\text{THz}$$.

In Fig. 12a, based on (5), we depict the real and imaginary parts of the permittivities with respect to operational frequency $\omega /(2\pi )$; note that $\text{Im}(\varepsilon _p)$ is very low and, accordingly, no off-diagonal elements emerge. As far as the InAs is concerned, we adopt the model (4) with $\omega _p/(2\pi )\cong 89.2~\text{THz}$ and the rest of the parameters ($\omega _c$, $\Gamma$) same as in Fig. 9a. In addition, the nonreciprocity of the gyrotropic medium is weak and, similarly to Fig. 9a, the vertical dashed line denotes the frequency at which $\text{Re}(\varepsilon _t)=-\text{Re}(\varepsilon _p)=2$ where the proposed concept is usually implemented. In Fig. 12b, we represent the metric $\Delta \tau$ as a function of $\omega /(2\pi )$ for various static magnetic fields $B_0$. One directly observes that exactly at the aforementioned frequency $\omega /(2\pi )\cong 28~\text{THz}$, all the curves of nonreciprocity indicator reach a peak; in addition, as happens in Fig. 11a, the maximal value of $\Delta \tau$ is proportional to bias $B_0$.

**Figure 13:**
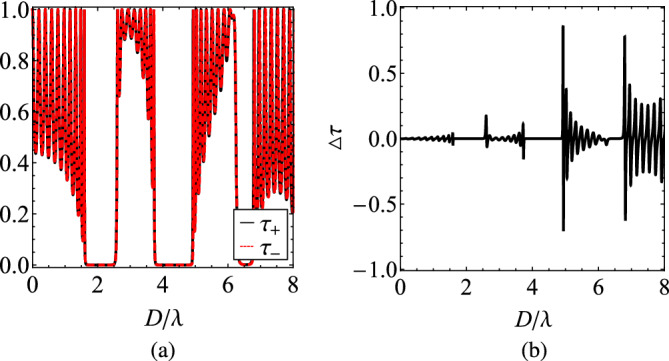
(**a**) The transmissivities $\tau _{\pm }$ and (**b**) the nonreciprocity indicator $\Delta \tau _{\pm }$ as functions of the overall thickness of the setup *D* normalized by the operational wavelength $\lambda$. Plot parameters: $N=10$, $r=1/2$, $\varepsilon _p=12$, $\varepsilon _{t}=2$, $\varepsilon _{g}=0.012$.

### Multiple scattering approach

Another strategy to enhance the nonreciprocal response without trying to suppress the diagonal elements of the effective matrix $[\varepsilon _\text{eff}]$ in (3) so that the off-diagonal ones dominate, may be based on maximizing multiple scattering. Indeed, when a circularly polarized wave illuminates a periodic multilayered system consisting of MO and dielectric layers, the heterostructure exhibits photonic bandgaps, namely, frequency bands across which the electromagnetic waves cannot propagate. At the boundaries of these bandgaps, we record strong reflections that are added to the propagating signals to form standing-wave patterns with group velocity close to zero. As shown above, gyrotropic media support two different propagating modes $k_{\pm }$ which, with the proper phase difference can give different transmissivities $\tau _{\pm }$ due to destructive and constructive interferences.

More specifically, we consider a structure similar to the one shown in Fig. 1, and replace the plasmonic layers by dielectric ones of positive permittivity $\varepsilon _p>0$. In Fig. 13a we depict the transmissivities $\tau _{\pm }$ as functions of optical thickness $D/\lambda$ for $N=10$ cells. Obviously, for a sufficiently large *N*, the considered heterostructure mimics the infinite periodic multilayers; thus, certain bandgaps for $D/\lambda$ emerge, outside of which the responses vary abruptly. Hence, only slightly different curves of the two transmissivities $\tau _{\pm }$ can lead to ultra-sharp maxima for $|\Delta \tau |$. Such a result is demonstrated by Fig. 13b where the nonreciprocity indicator becomes more pronounced close to the limiting values of $D/\lambda$ that define each bandgap. It is also important to stress that with this, plasmonic-free, approach all peaks of $|\Delta \tau |$ (even the weaker ones) are extremely narrowband which was not always the case in the rest of our results. Another indication that following the strategy to suppress the diagonal elements of the effective permittivity matrix is multiply advantageous.

### Overview

To sum up, built-in nonreciprocity of materials is usually weak and even when directional preference gets artificially imposed via static or phase-modulated biases, several challenges like high cost, increased risk or fabrication difficulties may appear. In this work, we propose a concept of significantly boosting the nonreciprocity of any gyrotropic or magneto-optical substance by using it in the form of multilayers, hosted by an ordinary epsilon-negative material. This placement of our gyrolayers into a plasmonic “sea” creates a new medium whose effective permittivity tensor has similar off-diagonal elements to the magneto-optical material, since plasmonics are isotropic. On the contrary, the diagonal permittivity elements of the considered heterostructure can be suppressed close to zero and thus make the off-diagonal ones, determining the nonreciprocity, look giant. In this way, the nonreciprocal nature of the suggested effective medium will be substantially enhanced while the transmissivity can be controlled via the thickness of the cavity.

The introduced idea has been validated by solving rigorously the formulated boundary value problem and demonstrating huge amplification in the nonreciprocity especially in the vicinity of bandgaps that emerge in the operation of the layered structure. As expected, the number of unit cells make the nonreciprocal resonances sharper and more pronounced while dissipation harms only moderately the reported enhancement. The proposed concept has been also tested in the presence of materials dispersion and it is shown that nonreciprocity is boosted when using indium arsenide or silicon carbide across the mid-infrared spectrum. Therefore, the examined heterostructures are both efficient and realistic, able to be incorporated in photonic integrating systems calling for nonreciprocity, from isolators and circulators to optical diodes and transistors.

## Methods

To solve the boundary value problem of the considered multilayers shown in Fig. 1 and determine the unknown fields across the structure, we follow the standard transfer-matrix formalism^50^. We assume one-dimensional propagation of RCP and LCP waves via a slab of thickness *h* filled by an anisotropic medium 1 with impedances $Z_1^{\pm }$ and wavenumbers $k_1^{\pm }$ that meets normally a region filled by another anisotropic medium 2 with impedances $Z_2^{\pm }$. The complex magnitudes of the four developed waves (transmission for RCP/LPC waves and reflection for RCP/LPC waves) into the first area are written as the respective coefficients into the second region multiplied by a $4\times 4$ transfer matrix ${\textbf {M}}_h\left( Z^{\pm }_1,Z^{\pm }_2,k^{\pm }_1\right)$.

This key matrix is written as the product of a block diagonal matrix expressing the impedance contrast and a diagonal matrix fixing the phases of the corresponding waves as they propagate through the layer of medium 1 and thickness *h*, namely:

6 $$\begin{aligned} {\textbf {M}}_h\left( Z^{\pm }_1,Z^{\pm }_2,k^{\pm }_1\right) = \left[ \begin{array}{cc} {\textbf {A}}(Z^{\pm }_1,Z^{\pm }_2) &{} {\textbf {B}}(Z^{\pm }_1,Z^{\pm }_2) \\ {\textbf {B}}(Z^{\pm }_1,Z^{\pm }_2) &{} {\textbf {A}}(Z^{\pm }_1,Z^{\pm }_2) \end{array}\right] \cdot \text{diag}\left( e^{+ik_1^{+}h},e^{+ik_1^{-}h},e^{-ik_1^{+}h},e^{-ik_1^{-}h}\right) . \end{aligned}$$

The submatrices ${\textbf {A}}$ and ${\textbf {B}}$ are given by:

7 $$\begin{aligned} {\textbf {A}}(Z^{\pm }_1,Z^{\pm }_2)= \left[ \begin{array}{cc} \frac{Z_1^{+}}{Z_2^{+}}\frac{Z_1^{-}+Z_2^{+}}{Z_1^{+}+Z_1^{-}} &{} 0 \\ 0 &{} \frac{Z_1^{-}}{Z_2^{-}}\frac{Z_1^{+}+Z_2^{-}}{Z_1^{+}+Z_1^{-}} \end{array}\right] ~~~~~~~,~~~~~~~ {\textbf {B}}(Z^{\pm }_1,Z^{\pm }_2)= \left[ \begin{array}{cc} 0 &{} \frac{Z_1^{+}}{Z_2^{-}}\frac{Z_1^{-}-Z_2^{-}}{Z_1^{+}+Z_1^{-}} \\ \frac{Z_1^{-}}{Z_2^{+}}\frac{Z_1^{+}-Z_2^{+}}{Z_1^{+}+Z_1^{-}} &{} 0 \end{array}\right] . \end{aligned}$$

These matrices incorporate the necessary boundary conditions along the interface between medium 1 and medium 2.

If we apply the considered transformation at the front surface of our multilayer system (see Fig. 1), the complex amplitudes of the incident and reflective fields into vacuum are expressed in terms of the respective ones of the developed eigenwaves into the first gyrotropic layer via the matrix ${\textbf {M}}_0\left( \eta _0,Z^{\pm },k_0\right)$ of zero thickness ($h=0$) that takes into account only the textural discontinuity. Similarly, the influence of each cell onto the local fields is expressed via the product of two transfer matrices: one describing the transformation due to a gyrotropic slab of size $h=(1-r)d$ into our plasmonic medium, namely, ${\textbf {M}}_{(1-r)d}\left( Z^{\pm },\frac{\eta _0}{\sqrt{\varepsilon _{p}}},k^{\pm }\right)$ and another representing the effect of the propagation into the plasmonic layer with thickness $h=rd$ before meeting a gyrotropic region, namely, ${\textbf {M}}_{rd}\left( \frac{\eta _0}{\sqrt{\varepsilon _{p}}},Z^{\pm },k_0\sqrt{\varepsilon _{p}}\right)$. Finally, after *N* cells, the last gyrolayer ($h=(1-r)d$) and the rear boundary with vacuum, where the transmission occurs, is involved by the transfer matrix ${\textbf {M}}_{(1-r)d}\left( Z^{\pm },\eta _0,k^{\pm }\right)$.

In this way, a $4\times 4$ linear system with respect to $\{R_{\pm },T_{\pm }\}$ is formulated as in:

8 $$\begin{aligned} {\textbf {r}}= {\textbf {M}}_0\left( \eta _0,Z^{\pm },k_0\right) \cdot \left\{ {\textbf {M}}_{(1-r)d}\left( Z^{\pm },\frac{\eta _0}{\sqrt{\varepsilon _{p}}},k^{\pm }\right) \cdot {\textbf {M}}_{rd}\left( \frac{\eta _0}{\sqrt{\varepsilon _{p}}},Z^{\pm },k_0\sqrt{\varepsilon _{p}}\right) \right\} ^N \cdot {\textbf {M}}_{(1-r)d}\left( Z^{\pm },\eta _0,k^{\pm }\right) \cdot {\textbf {t}}, \end{aligned}$$

where $${\textbf {r}}=[\begin{array}{cccc} 1&1&R_{+}&R_{-}\end{array}]^\text{T}$$ and $${\textbf {t}}=[\begin{array}{cccc} T_{+}&T_{-}&0&0 \end{array}]^\text{T}$$ are the vectors for the unknown reflection and transmission coefficients respectively, as they appear at (2). Therefore, the transmissivities $(\tau _{+},\tau _{-})$ are rigorously found and the considered metric $\Delta \tau =\tau _{+}-\tau _{-}$ can be directly evaluated for an arbitrary assortment of structural (optical footprint of the layout $D/\lambda$ and number of cells *N*), textural (employed gyrotropic and plasmonic materials) or excitation (RCP and LCP impinging waves) parameters.

It is important to stress that the whole $4\times 4$ S-parameters matrix of the setup depicted in Fig. 1 is written in terms only of $\left\{ T_{\pm },R_{\pm }\right\}$ as follows:

9 $$\begin{aligned} {\textbf {S}}=\left[ \begin{array}{cccc} 0 &{} R_{+} &{} T_{+} &{} 0 \\ R_{-} &{} 0 &{} 0 &{} T_{-} \\ T_{-} &{} 0 &{} 0 &{} R_{+} \\ 0 &{} T_{+} &{} R_{-} &{} 0 \end{array}\right] , \end{aligned}$$

where the first two lines concern the left port (RCP and LCP waves) and the last two are referred to right port (RCP and LCP waves). Note that once we impose a RCP wave at one side, the reflection is only of LCP type and vice-versa; thus, the diagonal of (9) contains only zeros. It is well-known that nonreciprocity appears in a network as long as ${\textbf {S}}\ne {\textbf {S}}^\text{T}$. For this reason, a good metric for the degree of nonreciprocity of a device^61^ can be the norm of the matrix $\Delta {\textbf {S}}=\left[ |S_{ml}|^2-|S_{lm}|^2\right]$, for $l,m=1,\cdots , 4$. Such a quantity $\left\| \Delta {\textbf {S}}\right\|$ is directly proportional to $\Delta \tau \equiv |T_{+}|^2-|T_{-}|^2$, given the symmetry of the considered structure imposing that: $|R_{+}|^2=|R_{-}|^2$.

## Data Availability

The datasets used and/or analysed during the current study available from the corresponding author on reasonable request.
